# Characterisation of a Plancitoxin-1-Like DNase II Gene in *Trichinella spiralis*

**DOI:** 10.1371/journal.pntd.0003097

**Published:** 2014-08-28

**Authors:** Chengshui Liao, Mingyuan Liu, Xue Bai, Pan Liu, Xuelin Wang, Tingting Li, Bin Tang, He Gao, Qingsong Sun, Xidong Liu, Ying Zhao, Feng Wang, Xiuping Wu, Pascal Boireau, Xiaolei Liu

**Affiliations:** 1 Key Laboratory for Zoonosis Research, Ministry of Education, Institute of Zoonosis, Jilin University, Changchun, People's Republic of China; 2 Jiangsu Co-innovation Center for Prevention and Control of Important Animal Infectious Diseases and Zoonoses, Yangzhou, People's Republic of China; 3 National Institute of Parasitic Diseases, Chinese Center for Disease Control and Prevention, Shanghai, People's Republic of China; University of Melbourne, Australia

## Abstract

**Background:**

Deoxyribonuclease II (DNase II) is a well-known acidic endonuclease that catalyses the degradation of DNA into oligonucleotides. Only one or a few genes encoding DNase II have been observed in the genomes of many species. 125 DNase II-like protein family genes were predicted in the *Trichinella spiralis* (*T. spiralis*) genome; however, none have been confirmed. DNase II is a monomeric nuclease that contains two copies of a variant HKD motif in the N- and C-termini. Of these 125 genes, only plancitoxin-1 (1095 bp, GenBank accession no. XM_003370715.1) contains the HKD motif in its C-terminus domain.

**Methodology/Principal Findings:**

In this study, we cloned and characterised the plancitoxin-1 gene. However, the sequences of plancitoxin-1 cloned from *T. spiralis* were shorter than the predicted sequences in GenBank. Intriguingly, there were two HKD motifs in the N- and C-termini in the cloned sequences. Therefore, the gene with shorter sequences was named after plancitoxin-1-like (*Ts*-Pt, 885 bp) and has been deposited in GenBank under accession number KF984291. The recombinant protein (r*Ts*-Pt) was expressed in a prokaryotic expression system and purified by nickel affinity chromatography. Western blot analysis showed that r*Ts*-Pt was recognised by serum from *T. spiralis*-infected mice; the anti-r*Ts*-Pt serum recognised crude antigens but not ES antigens. The *Ts*-Pt gene was examined at all *T. spiralis* developmental stages by real-time quantitative PCR. Immunolocalisation analysis showed that *Ts*-Pt was distributed throughout newborn larvae (NBL), the tegument of adults (Ad) and muscle larvae (ML). As demonstrated by DNase zymography, the expressed proteins displayed cation-independent DNase activity. r*Ts*-Pt had a narrow optimum pH range in slightly acidic conditions (pH 4 and pH 5), and its optimum temperature was 25°C, 30°C, and 37°C.

**Conclusions:**

This study indicated that *Ts*-Pt was classified as a somatic protein in different *T. spiralis* developmental stages, and demonstrated for the first time that an expressed DNase II protein from *T. spiralis* had nuclease activity.

## Author Summary

Deoxyribonuclease II (DNase II) is classified into a unique family of nucleases and mediates the degradation of DNA associated with apoptosis. Although DNase II activity was first observed in 1947, and has been studied biochemically and enzymatically since the 1960s, only recently has genetic information on the enzyme been reported. Compared with enzymes from other species, including *C. elegans*, the DNase II-like protein family of the parasitic nematode *T. spiralis* has expanded remarkably, with an estimated 125 genes found in the draft genome of *T. spiralis*. However, none of these proteins have been confirmed by biochemical studies. This study describes *Ts*-Pt, a DNase II protein that is expressed in different *T. spiralis* developmental stages. The recombinant protein purified via a prokaryotic expression system displayed *in vitro* nuclease activity, as determined by DNase zymography. The exact function and mechanisms of *Ts*-Pt should be further explored *in vivo*.

## Introduction

Deoxyribonucleases (DNases) are typically divided into two distinct categories, namely, Deoxyribonuclease I (DNase I) and Deoxyribonuclease II (DNase II), based their biochemical properties during DNA degradation [1]. There are also many different subclasses. DNase II (EC 3.1.22.1) is a well-known acid endonuclease that catalyses the dissection of DNA molecules into oligonucleotides by single-strand nicking and a double-strand cleavage mechanism [2]. DNase II generates 5′-hydroxyl groups and 3′-phosphate groups without divalent metal ions, but DNase I requires divalent metal ions for its catalytic activity and produces 5′-phosphate groups and 3′-hydroxyl groups [2]. DNase II activity was first observed in 1947 [3], and many studies have biochemically characterised these enzymes in mammalian systems [4], [5]. However, the nucleotide and amino acid sequences of these genes were unclear until the human DNase II gene was cloned in 1998 [6]. Soon after this initial report, DNase II or DNase II homologues were identified in vertebrates, invertebrates, and non-metazoans [7].

*Trichinella spiralis* (*T. spiralis*) is an intracellular pathogen of skeletal muscle and one of the most widespread zoonotic parasitic nematodes in the world [8]. It is especially prevalent in China, Argentina, and some eastern European countries [9]. To date, eight species and four genotypes have been classified in the genus *Trichinella*. The complete basic life cycle in a single host includes adult worms (Ad), newborn larvae (NBL), and muscle larvae (ML). Approximately 11 million people in 55 countries carry the infection, which is transmitted by eating of poorly cooked or raw infected meat [8]; the infection has a 0.2% mortality rate [9]. Trichinellosis is not only a serious public health threat but also an important economic factor in animal production and food safety [9].

Compared with enzymes from other species including *C. elegans*, the DNase II-like protein family in *T. spiralis* has expanded remarkably, with an estimated 125 genes in the genome [10]. Based on comparative protein sequence analyses, around half of these genes encode excretory-secretory (ES) products that are implicated in host-parasite interactions, and these proteins have been suggested as vaccine candidates for the control and prevention of trichinellosis [11]. A histidine residue that is surrounded by a highly conserved 5-mer, DHSKW [12], has been proposed as the core catalytic centre of most DNase II family members [13]. Of the 125 genes predicted as the DNase II-like protein family in *T. spiralis*, only plancitoxin-1 (1095 bp, GenBank accession no. XM_003370715.1) possesses one predicted active site involved in DNA cleavage and located in C-terminus. To date, neither its expression nor its activity has been explored. In the present study, we cloned and characterised the plancitoxin-1 gene from *T. spiralis*. The cloned sequences were shorter than the predicted sequences in GenBank and named after plancitoxin-1-like (*Ts*-Pt, 885 bp, GenBank accession no. KF984291). Meanwhile, the DNase activity of the recombinant *Ts*-Pt protein (r*Ts*-Pt) was examined.

## Methods

### Parasites

Parasites were prepared from different stages of the *T. spiralis* (ISS534) life cycle, as previously described [14]. Briefly, mice were experimentally infected per os with 400 L1 infective larvae, and *T. spiralis* ML were recovered at 35 and 60 days post-infestation (dpi) using a standard pepsin-hydrochloric acid digestion method. Rats were experimentally infected per os with 6000 L1 infective larvae, and Ad were isolated from the small intestines at 1 (Ad1), 3 (Ad3), and 6 dpi (Ad6). NBL were obtained from female Ad at 6 dpi and were incubated overnight in RPMI-1640 medium (Gibco, USA) containing 200 U/mL penicillin (Sigma, USA) and 200 µg/mL streptomycin (Sigma, USA) at 37°C and 5% CO_2_.

### Animals and ethics statement

BALB/c mice (18±2 g) and Wistar rats (180±20 g) aged 6–8 weeks and New Zealand white rabbits (2±0.2 kg) aged 12 weeks were obtained from the Experimental Animal Centre of College of Basic Medical Sciences, Jilin University (Changchun, China). Animals were free of specific pathogens and were housed and fed in compliance with the National Institutes of Health guidelines (publication no. 85-23, revised 1996). Animals were reviewed and approved by the Ethical Committee of Jilin University affiliated to the Provincial Animal Health Committee, Jilin Province, China (Ethical Clearance number IZ-2009-008).

### RNA isolation, cDNA synthesis, cloning, and real-time quantitative PCR

Total RNA from ML, Ad and NBL was extracted using a Trizol RNA extraction kit (Invitrogen, USA) and transcribed into first-strand cDNA with a SuperScript II RT cDNA synthesis kit (Invitrogen, USA), according to the manufacturer's instructions. The transcription levels for *Ts*-Pt were evaluated in different *T. spiralis* developmental stages with a forward primer (5′-*GAATAATACTGTCAACTGGAAT*-3′), reverse primer (5′-*TTTAGGAATGCTGTGAATTAG*-3′) and SYBR Premix Ex Taq II (Tli RNaseH Plus) (TAKARA, China). Real-time quantitative PCR was performed on an ABI Prism 7500 sequence detection instrument (Applied Biosystems, Inc.), as previously described [15]. The housekeeping gene *GAPDH* (glyceraldehyde-3-phosphate dehydrogenase, GenBank accession no. AF452239) was amplified with the forward primer 5′-*GCTCCTATGTTGGTTATGGG*-3′ and the reverse primer 5′-*TTTGGGTTGCCGTTGTAG*-3′. The relative expression of *Ts*-Pt in different developmental stages was determined using the 2^−ΔCt^ method. Three independent experiments were performed.

### Sequence and phylogenetic analysis

All of nucleotide and amino acid sequences in this study were from the National Center for Biotechnology Information (NCBI) (http://www.ncbi.nlm.nih.gov/). DNAMAN (version 6.0.3.48) was used to analyse the homology between *Ts*-Pt and other 124 predicted DNase II-like protein family genes. Conserved domains were predicted at http://www.ncbi.nlm.nih.gov/Structure/cdd/wrpsb.cgi. The molecular weight and theoretical pI were analyzed by the software ProtParam from ExPASy (http://web.expasy.org/protparam/). Online softwares were used to analyze the rare codon (http://people.mbi.ucla.edu/sumchan/caltor.html) and recombinant protein solubility (http://biotech.ou.edu/). Signal peptide, transmembrane domain, and N-linked glycosylation sites were predicted by the SignalP program, TMHMM program, and NetNGlyc program, respectively (http://www.cbs.dtu.dk/services/). Multiple sequence alignments for DNase II protein families from various organisms was performed with CLASTALX (version 2.1). Phylogenetic analysis of amino acid sequences was carried out using PHYLIP (version 3.695). And a neighbor joining tree was generated by bootstrap analysis with 1000 replicates using PHYLIP-NEIGHBOR. Then, the phylogenetic tree was visualized and edited using FigTree (version 1.3.1).

### Expression and purification of recombinant protein

Forward (5′-*TTTTGGATCCATGGACGCACGTCGGCCGGTAT*-3′, *Bam*HI site underlined) and reverse primers (5′-*CCCAAGCTTTCAATATGGTGGAATAGGACAAAGT*-3′, *Hind*III site underlined) were used to amplify the *Ts*-Pt gene from cDNA and genomic DNA from larvae. The recombinant plasmid was constructed from a linearised pET-28a (+) expression vector (Novagen, Germany) and the target gene. DNA sequencing was performed on an automated DNA sequencer. r*Ts*-Pt was expressed from an *E. coli* Rosetta (DE3) strain after induction with 1 mM isopropyl-β-D-thiogalactopyranoside (IPTG), and the protein was purified by affinity chromatography using a His-Trap purification kit (GE, USA), per the manufacturer's instructions.

### Infected mice sera and rabbit antisera

Infected sera were collected at 35 dpi from BALB/c mice infected experimentally per os with 400 L1 infective *T. spiralis* larvae. The antisera against r*Ts*-Pt was produced in a rabbit injected subcutaneously with approximately 500 µg of purified r*Ts*-Pt mixed with complete Freund's adjuvant (FCA, Sigma, USA). Three additional booster injections containing 250 µg of r*Ts*-Pt mixed with incomplete Freund's adjuvant (IFA) was injected intradermally at 2-week intervals. Antibodies from blood serum were affinity purified using Protein A Sefinose (Sangon, China), according to the manufacturer's instructions. Affinity-purified antibodies were used for following western blotting and immunolocalisation experiments.

### SDS-PAGE and western blotting

r*Ts*-Pt, crude somatic extracts and excretory/secretory (ES) products of *T. spiralis* were subjected to SDS-PAGE on a 12% polyacrylamide gel and subsequently transferred to a nitrocellulose membrane (Millipore, USA). After blocking in TBST-B [25 mM Tris, pH 8.0, 125 mM NaCl, 0.05% Tween 20 (V/V), 3.7% BSA] for 2 h at 37°C or overnight at 4°C, the membrane was incubated with the primary antibodies (*T. spiralis*-infected mice serum and rabbit anti-r*Ts*-Pt serum) at a dilution of 1∶200 in TBST-B for 2 h at room temperature. Secondary antibodies conjugated to horseradish peroxidase (goat anti-rabbit IgG and goat anti-mouse IgG) (Dingguo, China) were diluted 1∶5000 in TBST-B and incubated with the membrane for 1 h at room temperature. The membrane was reacted with ECL (enhanced chemiluminescence) reagent (Pierce, USA) and exposed to BioMax film.

### SDS-PAGE zymography

A modified method described by Detwiler and Macintyre was used for SDS-PAGE zymography activity gels [16]. Briefly, an SDS-PAGE gel containing 50 µg/mL salmon sperm DNA (Sigma, USA) in the separation gel (12%) but not in the concentration gel (4%) was prepared. The samples were incubated in loading buffer without β-mercaptoethanol at 37°C for 15 min and electrophoresed at 4°C. After electrophoresis, the gels were shaken gently in 2.5% Triton X-100 (Sigma, USA) at 4°C for 30 min with 4 changes in buffer and subsequently rinsed in 50 mM sodium acetate (pH 5.4) reaction buffer. For the DNase reaction, the gels were incubated at 37°C for 36 h in reaction buffer. For the enzymatic property study, the gels were incubated in reaction buffer at different temperatures in the presence of metal ions and EDTA, with or without inhibitor, or in different pH buffer solutions. The gels were stained with ethidium bromide, visualised with UV light, and subsequently stained with Coomassie brilliant blue.

### Liquid chromatography tandem mass spectrometry (LC-MS/MS)

DNase bands were excised from SDS-PAGE zymography gels and stored in ultrapure water. LC-MS/MS was performed by ProtTech, Inc. (Phoenixville). Briefly, the sample was cleaned by washing with water and digested in-gel with trypsin in digestion buffer (100 mM ammonium bicarbonate, pH 8.5). The peptides were extracted with acetonitrile, completely dried, re-dissolved, and analysed by a NanoLC-ESI-MS/MS. The MS data were used to search against the non-redundant protein database (NR database, NCBI) with the ProTech ProtQuest software suite.

### Immunolocalisation

Immunostaining of worms was performed as described previously [17]. Briefly, whole *T. spiralis* ML, Ad and NBL were immersed in fixative solution (3.7% formaldehyde 10 min, cold 100% MeOH 5 min), permeabilised by incubation in PBS containing 1% Triton X-100 for 5 min and blocked with 3% BSA in PBST (PBS containing 0.1% Triton X-100). The worms were then incubated with rabbit anti-r*Ts*-Pt polyclonal antibody at 4°C overnight. Following 3 washes in PBST, the worms were incubated with Alexa Flour 594-labeled goat anti-rabbit IgG fluorescent antibody (Invitrogen, USA) at room temperature for 1 h. The worms were washed 3 times in PBST, stained with Hoechst 33342 (Invitrogen, USA) for 10 min, washed 3 additional times in PBST, mounted with 70% glycerol on slides, and observed under a fluorescence microscope.

### Statistical analysis

The transcription data were expressed as the means ± standard deviation (SD), and the differences among groups were analysed with a one-way ANOVA and Student's t-test. P values were denoted as follows: *p<0.05 and **p<0.01 (*p*<0.05 or less was considered statistically significant).

## Results

### Molecular characterisation of *Ts*-Pt

The *Ts*-Pt gene, comprising an-885 bp complete cds sequence, was obtained by PCR (data not shown), and has been deposited in GenBank (accession no. KF984291). The *Ts*-Pt sequence was 210 bp shorter than previously predicted plancitoxin-1 (Tsp_09974, GenBank accession no. XM_003370715.1). Sequence analysis revealed that the *Ts*-Pt gene encoded a protein of 294 amino acids with a predicted molecular mass (Mr) of 33.2 kDa and theoretical pI of 9.17 (http://web.expasy.org/protparam/). By sequence alignment, the *Ts*-Pt protein revealed less than 20% sequence identity to other 124 predicted DNase II-like protein family genes except AY790263 (22.13%), AY790264 (21.84%), AY790265 (21.18%), AY790266 (23.76%), Tsp_02430 (20.37%), Tsp_07454 (20.55%), Tsp_11476 20.57%), Tsp_11488 (20.06%), Tsp_11491 (21.90%), Tsp_11501 (20.56%), Tsp_12136 (22.95%), Tsp_12346 (20.29%), Tsp_12347 (21.84%).

**Figure 1:**
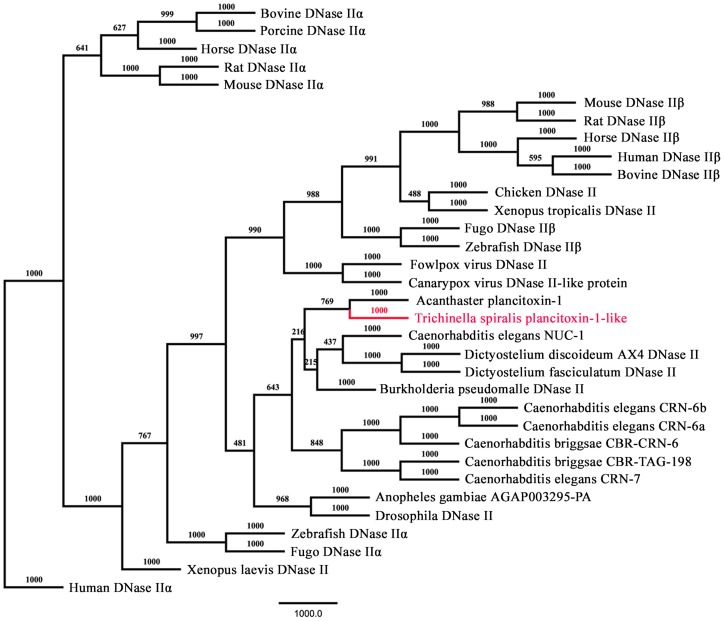
Phylogenetic analysis of *Ts*-Pt and other previously reported DNase II family proteins. Amino acid sequences of 33 DNase II were aligned by using CLASTALX 2.1 and phylogenetic tree was generated by the neighbor-joining method using PHYLIP 3.695. *Ts*-Pt was highlighted in red. The scale bar represents amino acid substitutions in the sequences and evolutionary distance.

Although the sequence homology of the *Ts*-Pt gene was quite low compared with genes from other species, the NCBI non-redundant protein sequence database indicated that the protein shared a deeper homology with DNase II or DNase II-like proteins from a wide variety of eukaryotic and prokaryotic species (data not shown). The phylogenetic relationships between *Ts*-Pt and previously reported DNase II family members were determined based on sequence similarities. A total of 33 DNase II sequences from various organisms (Text S1) including human, bovine, horse, porcine, mouse, rat, chicken, fugo, *Drosophila*, *Xenopus laevis*, *Xenopus tropicalis*, *Acanthaster*, zebrafish, *Anopheles gambiae*, *C. elegans*, *C. briggsae*, *Burkholderia pseudomalle*, *Dictyostelium fasciculatum*, *Dictyostelium discoideum*, canarypox virus, fowlpox virus, and *T. spiralis* were used to reconstruct the phylogenetic relationships. *Ts*-Pt and *Acanthaster* plancitoxin-1 belonged to the same clade (Fig. 1) and had the similarity with 41%. In addition, the protein contained four potential N-linked glycosylation sites (Asp-X-Thr/Ser) at positions 19, 49, 209 and 262. Neither a signal peptide nor a transmembrane domain was found in the derived amino acid sequence (http://www.cbs.dtu.dk/services/).

### SDS-PAGE and western blot analysis of *Ts*-Pt

**Figure 2:**
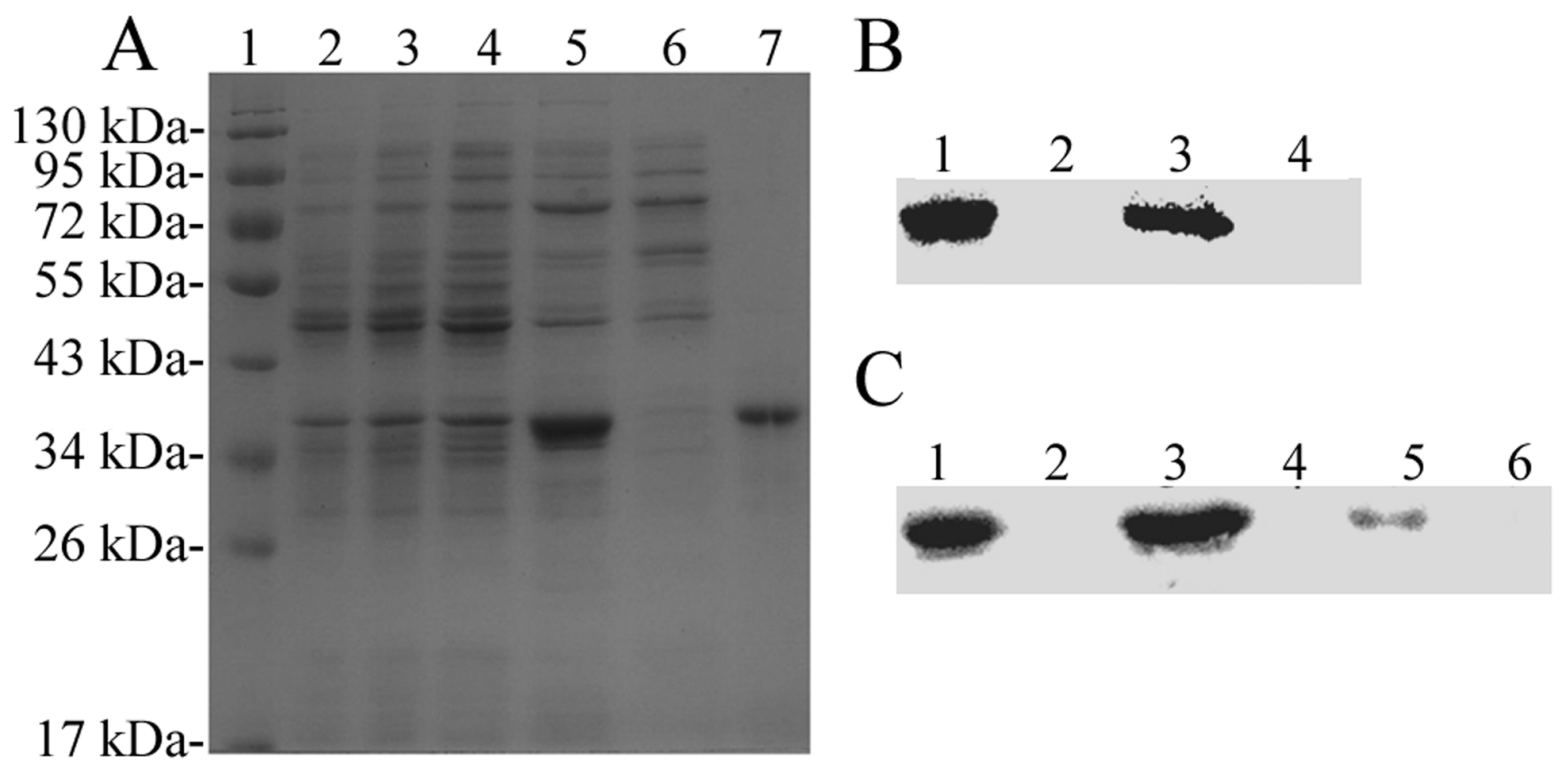
Identification of r*Ts*-Pt by SDS-PAGE and western blotting. (A) SDS-PAGE analysis of the expression and purification of r*Ts*-Pt. The gel was stained with Coomassie Brilliant blue. Lane 1, prestained protein marker (Genview); lane 2, *E. coli* lysate (pET28a) without IPTG; lane 3, *E. coli* lysate (pET28a) with IPTG; lane 4, *E. coli* lysate (pET-28a/*Ts*-Pt) without IPTG; lane 5, *E. coli* lysate (pET-28a/*Ts*-Pt) with IPTG; lane 6, supernatant of *E. coli* lysate (pET-28a/*Ts*-Pt) with IPTG; lane 7, r*Ts*-Pt purified by Ni-affinity chromatography. (B) Western blot analysis of the antigenicity of r*Ts*-Pt. Lane 1, rabbit anti-r*Ts*-Pt serum; lane 2, negative rabbit serum; lane 3, *T. spiralis*-infected mice serum; lane 4, negative mice serum. (C) Western blot analysis of the crude somatic extracts and ES products of *T. spiralis* were recognised by rabbit anti-r*Ts*-Pt serum. Lane 1, Ad crude somatic extracts; lane 2, Ad ES products; lane 3, NBL crude somatic extracts; lane 4, NBL ES products; lane 5, ML crude somatic extracts; lane 6, ML ES products.

Sequence analysis showed that the *Ts*-Pt nucleotide sequence contained 26 rare codons (http://people.mbi.ucla.edu/sumchan/caltor.html). An *E. coli* Rosetta (DE3) strain was used to express the target protein, which had an estimated zero percent chance of solubility when overexpressed in *E. coli* (http://biotech.ou.edu/). The recombinant protein of approximately 36 kDa was observed in inclusion bodies after induction with IPTG, and it appeared as a single band on SDS-PAGE after purification (Fig. 2A). Western blot analysis revealed that r*Ts*-Pt can be recognized by serum from r*Ts*-Pt immunized but not pre-immunized rabbit. Serum from *T. spiralis*-infected mice also showed reactivity to r*Ts*-Pt, which implied the potential antigenicity of native *Ts*-Pt (Fig. 2B). To determine whether *Ts*-Pt was expressed as a somatic or secretory protein, the crude somatic extracts and ES products of *T. spiralis* (Ad, NBL, and ML) were reacted with the antibody against r*Ts*-Pt. As shown in Fig. 2, the antibody against r*Ts*-Pt recognised *T. spiralis* crude somatic extracts but not ES products (Fig. 2C).

### Real-time quantitative PCR analysis

**Figure 3:**
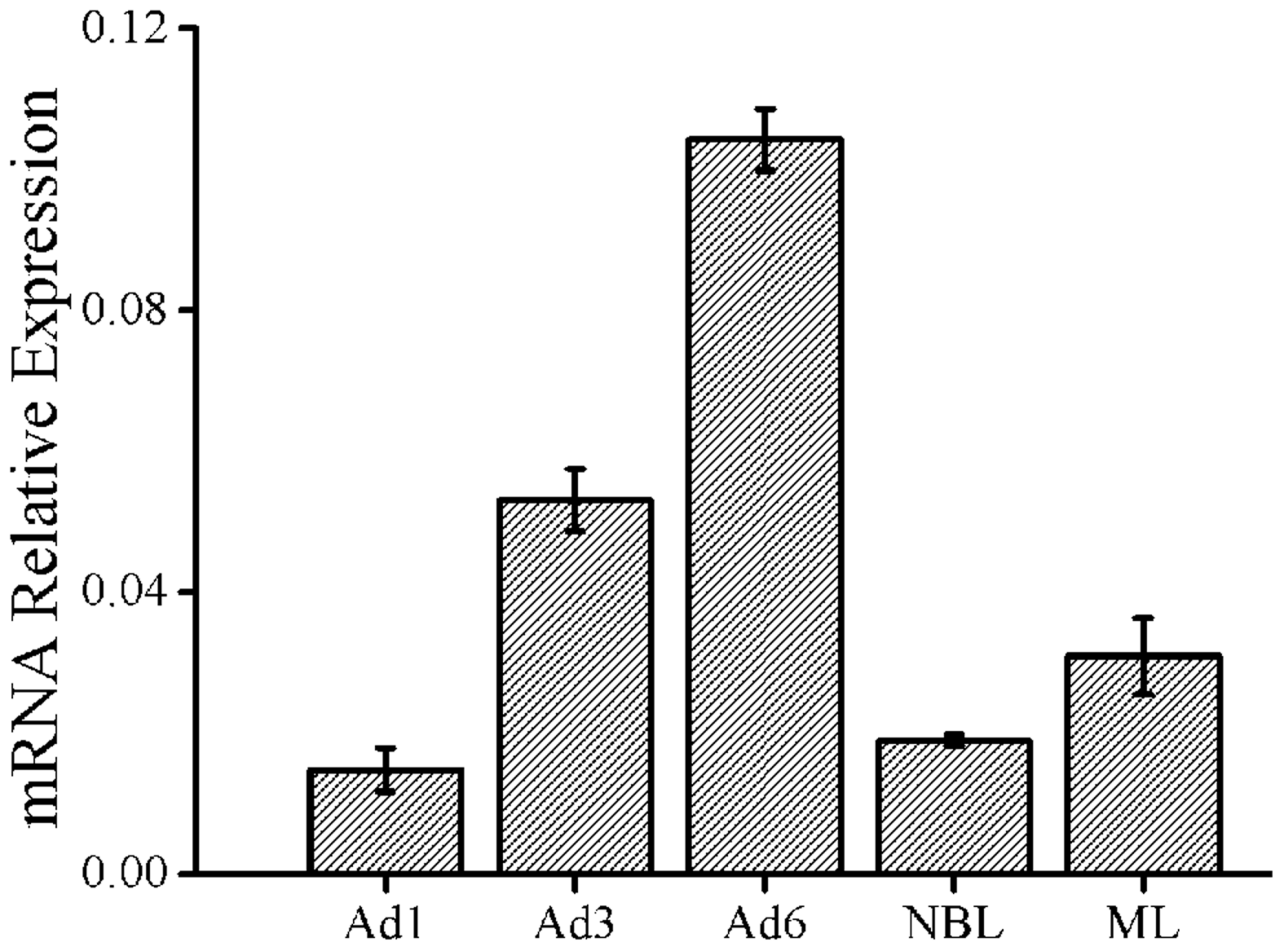
Real-time quantitative PCR detection of *Ts*-Pt mRNA at different developmental stages of *T. spiralis*. The relative expression of *Ts*-Pt mRNA was evaluated using the 2^−ΔCt^ method. GAPDH was used as an internal control. There were statistically significant differences (*p*<0.05) between any two groups other than Ad1 and NBL. Abbreviations: Ad1, adult worms at 1 days post-infestation; Ad3, adult worms at 3 days post-infestation; Ad6, adult worms at 6 days post-infestation; NBL, newborn larva; ML, muscle larva.

To quantify transcription of the *Ts*-Pt gene in different *T. spiralis* developmental stages, real-time quantitative PCR was performed. Although there were statistically significant differences in mRNA when any two groups other than Ad1 and NBL were compared, the *Ts*-Pt gene was expressed at all developmental stages (Fig. 3). In general, its expression in Ad1, Ad3, NBL, and ML was lower than in Ad6. For Ad, the expression of *Ts*-Pt mRNA rapidly increased to its maximum level at 6 dpi.

### Enzymatic properties of r*Ts*-Pt

**Figure 4:**
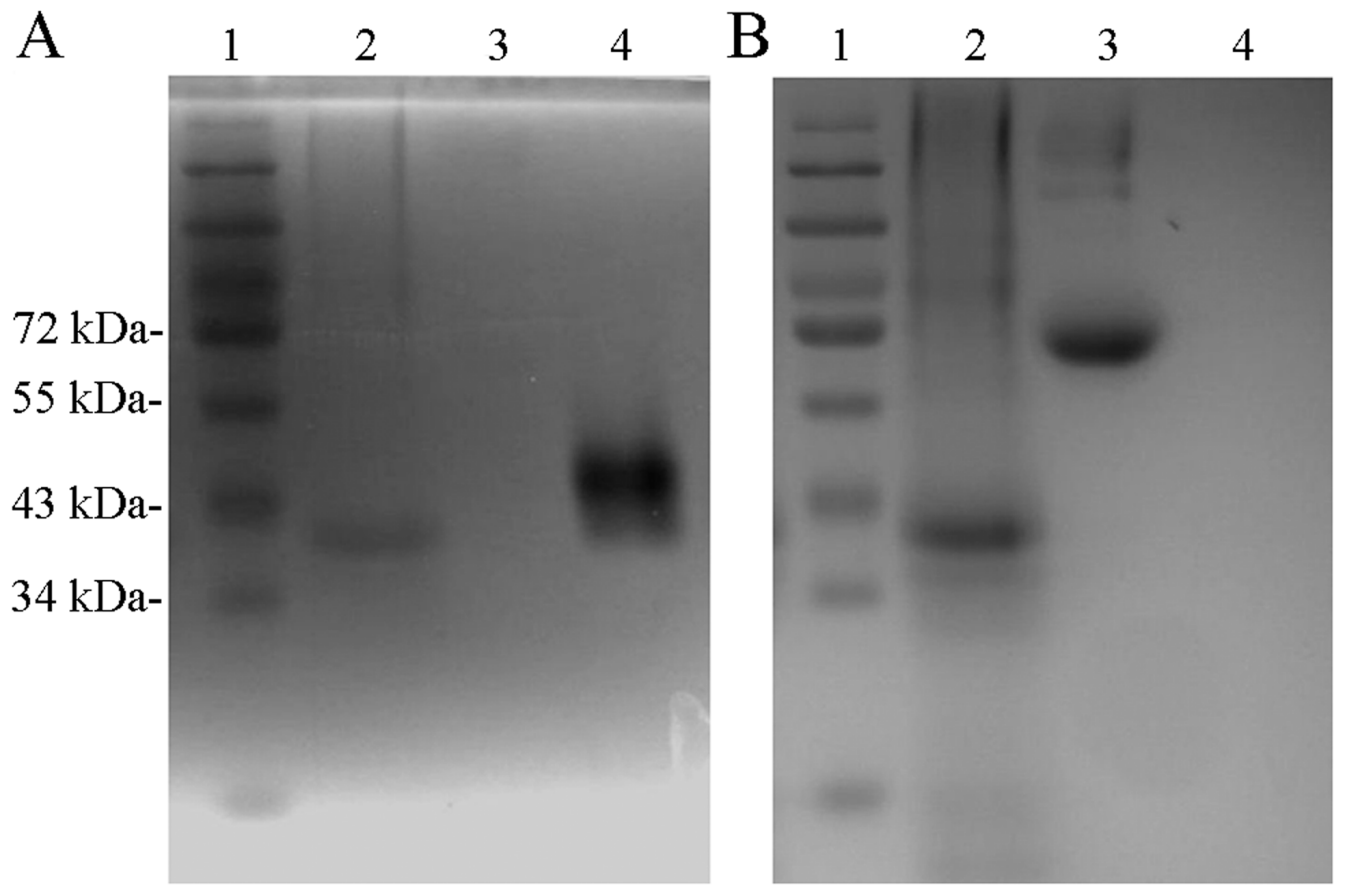
Detection of r*Ts*-Pt nuclease activity with SDS-PAGE zymography. (A) SDS-PAGE zymography analysis of r*Ts*-Pt, after staining with ethidium bromide and exposure to UV light. (B) The same gel was stained with Coomassie Brilliant blue after zymography. Lane 1, prestained protein marker (Genview, Houston, Texas); lane 2, r*Ts*-Pt; lane 3, BSA (negative control); lane 4, DNase II (positive control).

**Figure 5:**
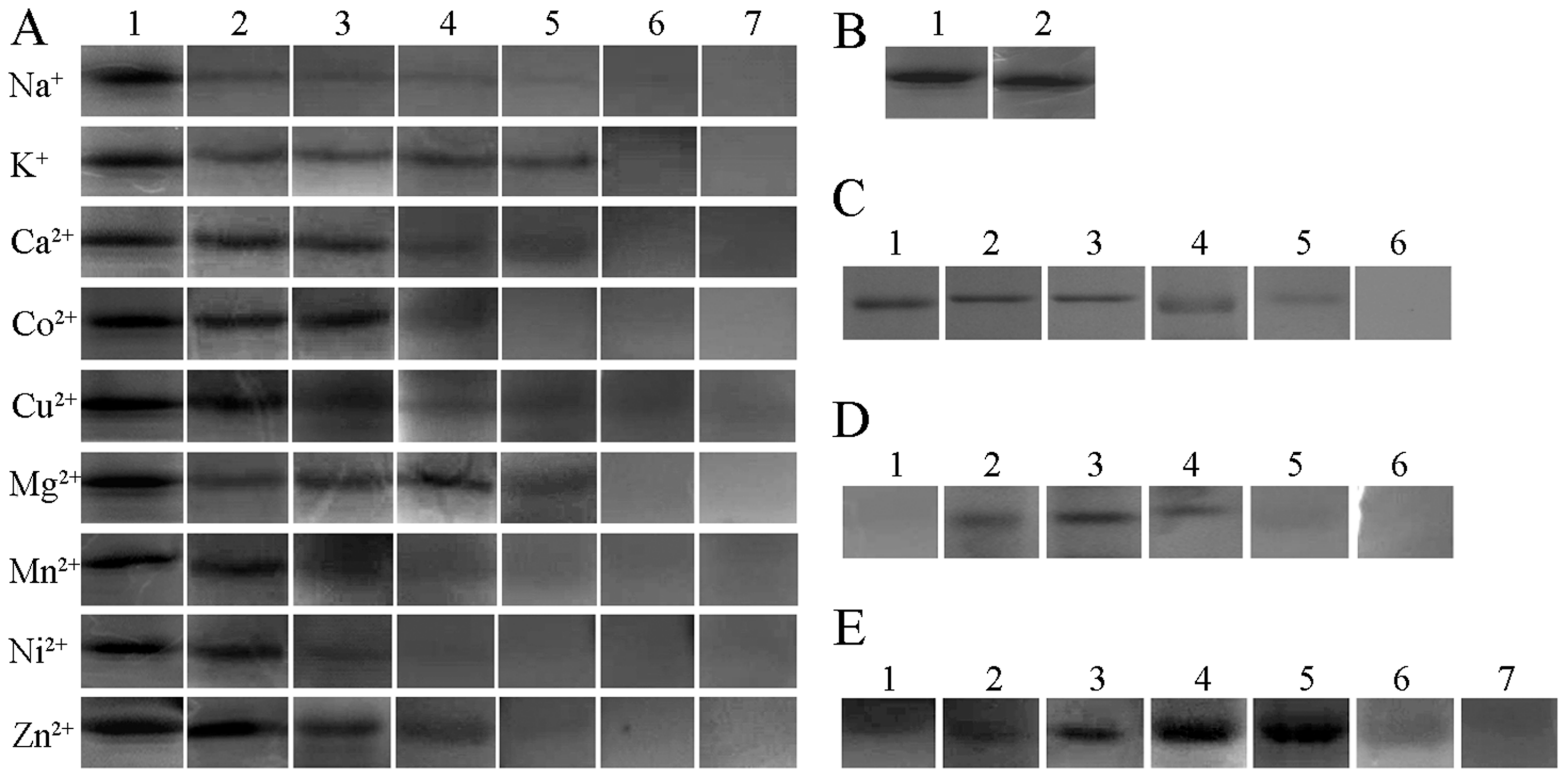
Detection of r*Ts*-Pt activity in various conditions using SDS-PAGE zymography. (A) SDS-PAGE zymography analysis of r*Ts*-Pt in the presence of various metal ions. The gel was stained with ethidium bromide. The numbers 1, 2, 3, 4, 5, 6, and 7 indicate the presence of 0 mM, 5 mM, 10 mM, 20 mM, 30 mM, 40 mM, and 50 mM metal ions, respectively. (B) SDS-PAGE zymography analysis of r*Ts*-Pt in the presence of 1 mM EDTA. Lane 1, normal; Lane 2, EDTA. (C) SDS-PAGE zymography analysis of r*Ts*-Pt in the presence of ATA. The gel was stained with ethidium bromide. The numbers 1, 2, 3, 4, 5, and 6 indicate the presence of 0 µM, 1 µM, 5 µM, 15 µM, 20 µM, and 25 µM ATA, respectively. (D) SDS-PAGE zymography analysis of r*Ts*-Pt in different pH conditions. The gel was stained with ethidium bromide. The numbers 1, 2, 3, 4, 5, and 6 indicate pH 3, pH 4, pH 5, pH 6, pH 7, and pH 8, respectively. (E) SDS-PAGE zymography analysis of r*Ts*-Pt in different temperatures. The gel was stained with ethidium bromide. The numbers 1, 2, 3, 4, 5, 6, and 7 indicate 16°C, 20°C, 25°C, 30°C, 37°C, 42°C, and 50°C, respectively.

*Ts*-Pt was predicted to have DNase II activity based on sequence homology. We determined the nuclease activity of *Ts*-Pt using r*Ts*-Pt protein purified by His affinity. r*Ts*-Pt protein catalysed the degradation of salmon sperm DNA in 50 mM sodium acetate and appeared as a single black band of ∼36 kDa under UV light (Fig. 4). To further examine the catalytic properties of r*Ts*-Pt, we analysed r*Ts*-Pt activity in conditions with various temperatures and pH and in the presence or absence of metal ions, EDTA, and nuclease inhibitors. In a narrow range of slightly acidic conditions, r*Ts*-Pt had it optimal nuclease activity, which disappeared in an alkaline environment at 37°C (Fig. 5D). The single black band was observed clearly at 25°C, 30°C, and 37°C in 50 mM sodium acetate, weakly observed at 20°C and 42°C, and not observed at 16°C or 50°C (Fig. 5E). High concentrations of metal ions inhibited r*Ts*-Pt activity at 37°C in 50 mM sodium acetate (Fig. 5A), and nuclease inhibitors and aurintricarboxylic acid (ATA, Sigma) had the same effect (Fig. 5C). Nuclease activity was not be affected by EDTA (Fig. 5B).

### LC-MS/MS analysis

The protein band was successfully identified by NanoLC-MS/MS. Four peptides were matched and characterised as *Ts*-Pt (Table 1). The MS data were only analysed by BLAST with the NCBI reference (accession no. XM_003370715.1) because the sequence submitted by us was not released.

**Table 1:** Protein identification of r*Ts*-Pt by NanoLC-MS/MS.*

Protein name	Mol. Mass (kDa)	No. matched peptides	Peptide Sequence	Accession No.
r*Ts*-Pt	41.694	4	FSDDVDFYFASDHSK	gi|316965711
			ELYVDLVAPTLK	
			FPLPTTFLYPNTGK	
			IPSLANIISGVQTVQPPYYSITK	

*After NanoLC-MS/MS analysis, the MS data was used to search against the most recent non-redundant protein database in NCBI.

### Immunolocalisation of *Ts*-Pt

**Figure 6:**
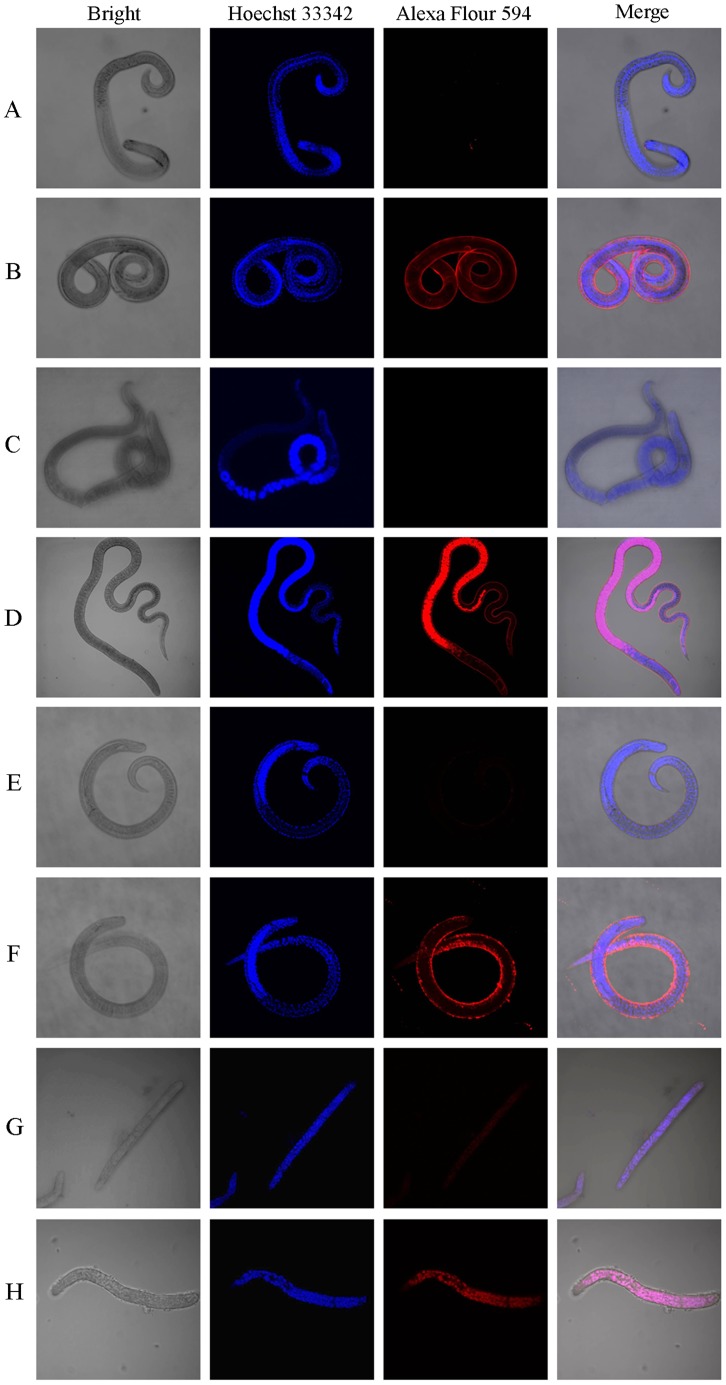
Immunolocalisation of *Ts*-Pt in the different developmental stages of *T. spiralis*. Intact whole Ad1 (A and B), Ad6 (C and D), ML (E and F), and NBL (G and F) were incubated with anti-r*Ts*-Pt rabbit serum (B, D, F, and H) or normal rabbit serum (A, C, E, and G). After incubation with the Alexa Fluor 594-labeled goat anti-rabbit IgG secondary antibody, the specimens were observed under a fluorescence microscope.

To evaluate the localisation of *Ts*-Pt in worms, immunofluorescence staining was performed. As shown in Fig. 6, strong red fluorescence signals were observed in the entire bodies of NBL and the teguments of Ad and ML after reaction with r*Ts*-Pt antibody. No fluorescence was observed in worms stained with the negative control rabbit serum.

## Discussion

**Figure 7:**
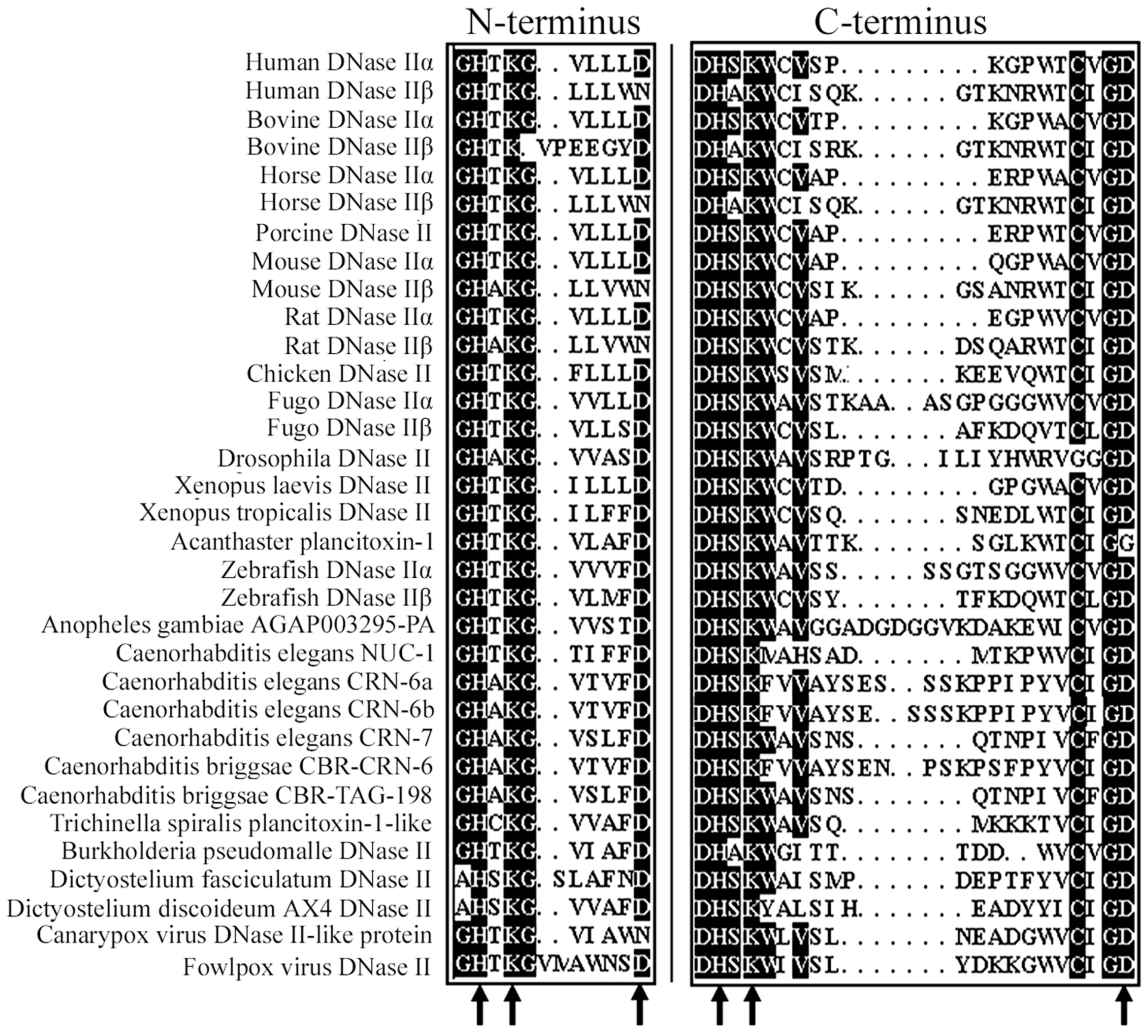
The catalytic domain of DNase II and similar proteins. DNase II contains two copies of a variant HKD motif, where the aspartic acid residue is variable; the motifs are distributed in both the N- and C-terminus domains (black arrows). The catalytic centre of DNase II is formed by the two variant HKD motifs in the form of a pseudodimer.

From the perspective of protein function, DNases are divided into three groups: the Mg^2+^-endonucleases, the Ca^2+^/Mg^2+^ endonucleases, and the cation-independent endonucleases. DNase II was discovered more than 50 years ago and belongs to the third group. Recently, DNase II has been purified, and its physical, molecular and enzymatic properties have been thoroughly examined. Three acidic DNase II enzymes—DNase IIα, DNase IIβ, and L-DNase II—have been identified since the human DNase II gene was first cloned in 1998 [18]. DNase II is a monomeric nuclease that contains two copies of a variant HKD motif (H-x-K-x(4)-D, where x represents any amino acid residue) in the N- and C-termini, suggesting a putative catalytic mechanism (Fig. 7). The two variant HKD motifs compose the catalytic centre of DNase II in a pseudodimeric way.

In *T. spiralis*, the endonuclease activity of at least three proteins was demonstrated in the excretory/secretory (ES) products [19]. p43, SS1, and AAK85403 have significant similarity to human DNase II [20]. Although the MYC-Tsp43 plasmid facilitated this expression as a recombinant protein in C_2_C_12_ myoblasts in the presence of the DNase inhibitor ATA [21], the *T. spiralis* protein responsible for nuclease activity is unknown. Of the 125 DNase II-like protein family genes in the *T. spiralis* genome, only plancitoxin-1 contains the HKD motif. In our work, the *Ts*-Pt gene encoded a protein of 294 amino acids, which is 70 amino acids shorter than the NCBI reference sequence of plancitoxin-1. Compared with plancitoxin-1, which has a variant HKD motif in the C-terminus domain, *Ts*-Pt had two motifs in the N- and C-terminus domains (data not shown). Generally, the putative active site sequences for DNase IIα and β are DHSK and DHAK, respectively, in the C-terminus domains [12]. However, mouse, rat, fugo, and zebrafish DNase IIβ enzymes contain a DHSK motif. Thus, it was difficult to classify *Ts*-Pt as a DNase IIα or DNase IIβ.

One method to identify potential nuclease activity is zymography. In this assay, DNA is incorporated into an SDS-polyacrylamide gel as a special substrate, and the loss of substrate from the gel matrix reflects nuclease activity [22]. Nuclease activity at a given molecular weight is recognised as a dark band of enzyme degradation in a white background in the gel under UV light, which is not stained by ethidium bromide. In previous studies, all proteins were obtained from animal, plant, bacteria, fungi, and parasite tissues and ES products. Soluble nucleases expressed via prokaryotic and eukaryotic expression systems can be used for nuclease zymography [23]. In this study, we have changed several expression parameters including temperature, IPTG concentration, induction time, bacterial host, expression vector, and medium but still failed to obtain soluble r*Ts*-Pt (data not shown). However, it is surprising that inclusion bodies could be used for nuclease zymography, showing limited degradation of DNA. The theoretical principle of this phenomenon cannot be elaborated completely in this paper; however, it is possible that the inclusion bodies were denatured by SDS and subsequently electrophoresed in SDS gels containing DNA. After electrophoresis, most misfolded proteins were allowed to renature in the gel by washing with 2.5% Triton X-100 to remove SDS. DNA was digested by the proteins that properly refolded in the reaction buffer.

*In vitro*, DNase II cuts DNA in 50 mM sodium acetate buffer (pH 4.6–5) with the addition of EDTA, and it exhibits weakly Ca^2+^/Mg^2+^-dependent endonuclease activity but strongly cation-independent endonuclease activity below pH 7 [24]. In this report, we showed that the nuclease r*Ts*-Pt was active in a narrow pH range (pH 4–5) and an optimum temperature range (25°C, 30°C, and 37°C). r*Ts*-Pt activity was suppressed by metal ions in concentrations greater than 20 mM, including K^+^, Na^+^, Ca^2+^, Co^2+^, Cu^2+^, Mg^2+^, Mn^2+^, Ni^2+^, and Zn^2+^. The chelator EDTA had no effect on r*Ts*-Pt activity, but a general nuclease inhibitor, ATA, affected r*Ts*-Pt activity.

Excluding *Xenopus tropicalis* DNase II, *Burkholderia pseudomalle* DNase II, *Dictyostelium fasciculatum* DNase II, canarypox virus DNase II-like protein, and fowlpox virus DNase II, all the proteins mentioned in Fig. 1 contain a signal peptide at the amino terminus, as predicted by the SignaIP 4.1 program analysis (http://www.cbs.dtu.dk/services/). For *T. spiralis*, no signal peptide or transmembrane domain was predicted in *Ts*-Pt. Western blotting showed that r*Ts*-Pt was expressed as a somatic protein in different developmental stages of *T. spiralis* and most highly expressed in Ad at 3 dpi. In vertebrates, DNase II, which is usually involved in various development processes and DNA degradation during cell death, is important for organismal homeostasis [7]. In *C. elegans*, there are three DNase II homologues, NUC-1, CRN-6 (K04H4.6), and CRN-7 (F09G8.2). These proteins play differential roles in apoptotic DNA degradation and development in *C. elegans*, and they have important regulative action in DNA degradation [25]. *Ts*-Pt was located in the entire bodies of NBL and the teguments of Ad and ML. It might remove damaged DNA during the growth and development of the parasite. Moreover, the tegument of the parasite is constantly in contact with the host cell. Thus, it may be associated with host-parasite interactions during infection.

In conclusion, we characterised a DNase II protein, *Ts*-Pt, which was expressed in the different developmental stages of *T. spiralis*. The recombinant protein was purified from a prokaryotic expression system, and it was determined to have nuclease activity *in vitro* via DNase zymography. To better understand the exact function and mechanisms of *Ts*-Pt *in vivo*, more detailed functional and mechanistic studies are needed.

## Supporting Information

Text S1 **GenBank accession numbers.**
(DOC)
