# Supplementary material for: Characterisation of a Plancitoxin-1-Like DNase II Gene in Trichinella spiralis
Source: PLoS Negl Trop Dis. 2014 Aug 28;8(8):e3097. doi: 10.1371/journal.pntd.0003097 (PMC4148230; doi:10.1371/journal.pntd.0003097)
Supplement: Text S1 — GenBank accession numbers. (DOC) [file pntd.0003097.s001.doc]

Previously reported DNase II family members: human DNase IIα (AF047016.1), human DNase IIβ (AF274571.1), bovine DNase IIα (NM_001075127.2), bovine DNase IIβ (NM_001075865.1), horse DNase IIα (XM_005611850.1), horse DNase IIβ (XM_005610562.1), porcine DNase II (AF060221.1), mouse DNase IIα (AF045741.1), mouse DNase IIβ (NM_019957.4), rat DNase IIα (NM_138539.2), rat DNase IIβ (NM_021664.1), chicken DNase II (DQ272298.1), fugo DNase IIα (XM_003965676.1), fugo DNase IIβ (XM_003974288.1), *Drosophila* DNase II (NM_142415.3), *Xenopus laevis* DNase II (NM_001093202.1), *Xenopus tropicalis* DNase II (NM_001011305.1), *Acanthaster* plancitoxin-1 (AB121229.1), zebrafish DNase IIα (NM_001114738.1), zebrafish DNase IIβ (XM_686359.5), *Anopheles gambiae* AGAP003295-PA (XM_319521.5), *C. elegans* NUC-1 (NM_077203.5), *C. elegans* CRN-6a (NM_066661.1), *C. elegans* CRN-6b (NM_066660.4), *C. elegans* CRN-7 (NM_066416.3), *C. briggsae* CBR-CRN-6 (XM_002641718.1), *C. briggsae* CBR-TAG-198 (XM_002641899.1), *Burkholderia pseudomalle* DNase II (EEP49373.1), *Dictyostelium fasciculatum* DNase II (XM_004367189.1), *Dictyostelium discoideum* AX4 DNase II (XM_630570.1), canarypox virus DNase II-like protein (NP_955074.1), and fowlpox virus DNase II (CAA07012.1) were taken from the NCBI GenBank in this paper. The nucleotide sequence of *T. spiralis* plancitoxin-1-like has been deposited in GenBank under accession number KF984291.
